# Endemic plants of Java Island, Indonesia: a dataset

**DOI:** 10.3897/BDJ.10.e84303

**Published:** 2022-07-07

**Authors:** Dipta Sumeru Rinandio, Hendra Helmanto, Rizmoon Nurul Zulkarnaen, Enggal Primananda, Arief Hamidi, Iyan Robiansyah

**Affiliations:** 1 Research Center for Plant Conservation, Botanic Gardens and Forestry, National Research and Innovation Agency, Bogor, Indonesia Research Center for Plant Conservation, Botanic Gardens and Forestry, National Research and Innovation Agency Bogor Indonesia; 2 Fauna & Flora International-Indonesia Programme, Jakarta, Indonesia Fauna & Flora International-Indonesia Programme Jakarta Indonesia

**Keywords:** ex situ conservation, Global Biodiversity Information Facility, IUCN Red List, plant growth forms, plant of the world online, trees

## Abstract

**Background:**

Java is the most populous island in the world. This high population and the extensive economic activities have significantly reduced the forest areas of the Island and have greatly increased the pressure on its plant diversity. Compared to those with a wide distribution, endemic plants with a narrow geographic range are more vulnerable to anthropogenic threats and environmental changes. As species lists are essential for knowledge of species diversity in areas with strong anthropogenic pressure, here we present a dataset of endemic plants of Java Island. The initial species list was manually extracted from the Plant of the World Online (POWO). Each species on the list was then confirmed for its endemism by checking its current distribution using peer-reviewed publications, online plant databases and herbarium specimen images stored on the Global Biodiversity Information Facility (GBIF). The dataset contains 652 species in 279 genera and 85 families. The family with the highest number of endemic species is Orchidaceae (142 species), followed by Rubiaceae (57 species), Acanthaceae (40 species), Apocynaceae (35 species) and Lauraceae (29 species). The growth form of the species is mostly trees (22.6%), followed by herbs (19.2%), epiphytes (16%), shrubs (12.4%), vines (11%) and geophytes (9.4%). Most of the species (89.7%) have not yet been assessed for their conservation status according to the IUCN Red List Categories and Criteria. There are only 55 species (8.3%) that have been conserved within ex situ collections. Furthermore, most of the species (79.8%) are not listed on the CITES appendices and there are only four species (0.6%) protected by national law.

**New information:**

Our contribution provides the first online list of accepted scientific names of Javan endemic plants species, together with all their synonyms. New to the dataset are: i) provision of local names of the species (if available), ii) the classification of species under eleven growth forms (tree, shrub, herb, annual, graminoid, geophyte, fern, vines, hydrophyte, parasite and epiphyte), iii) assignation of the extinction risk of species according to the IUCN Red List, iv) ex situ collection status of species and information on the protection status of the species according to (v) CITES and (vi) the national law of Indonesia.

## Introduction

Java is the most populous island in the world. With an area of just 7% of Indonesia’s total landmass, it has a population of 145 million or more than 56% of Indonesia’s total population (WorldAtlas 2021). This high population and the extensive economic activities have significantly reduced the forest areas of the Island and greatly increased the pressure on its plant diversity. Natural forests were estimated to cover 85% of Java in 1817 (Raffles 1817) and it dropped to only 8% in 1987 (BPS 1988). At the present time, the remaining original habitat of the Island is only about 5% (Morrison 2021). One endemic plant of the Island, *Etlingeraheyneana* (Valeton) R.M.Sm. (Zingiberaceae), has been declared to be extinct (Olander 2019) and the other 94 plant species are currently threatened with extinction (IUCN 2021). Pimm et al. (2014) stated that population growth and associated increasing consumption per capita are the main factors for species extinction.

Endemic plants are of great importance as they possess unique genetic materials and can provide basic needs and survival for the local people (Skarbek 2008). They, however, are more vulnerable to anthropogenic threats and environmental changes and, therefore, face a higher extinction risk. Therefore, the conservation of endemic plants is considered a global priority (Myers et al. 2000, Işik 2011). The following characteristics can usually be found in endemic plants that make them prone to extinction (Primack 2006):

1. narrow geographic range,
2. one or few populations,
3. small population size,
4. declining population size,
5. harvesting by people,
6. low reproductive and/or diaspora-dispersal ability and
7. specific habitat requirements.

To conserve the species and prevent them from extinction, a comprehensive conservation action that involves both in situ and ex situ programmes is urgently needed.

**Figure 1.**
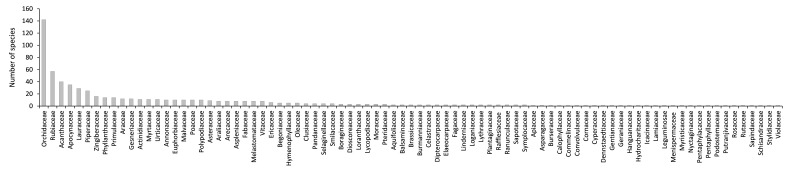
Species richness classified by number of families.

**Figure 2.**
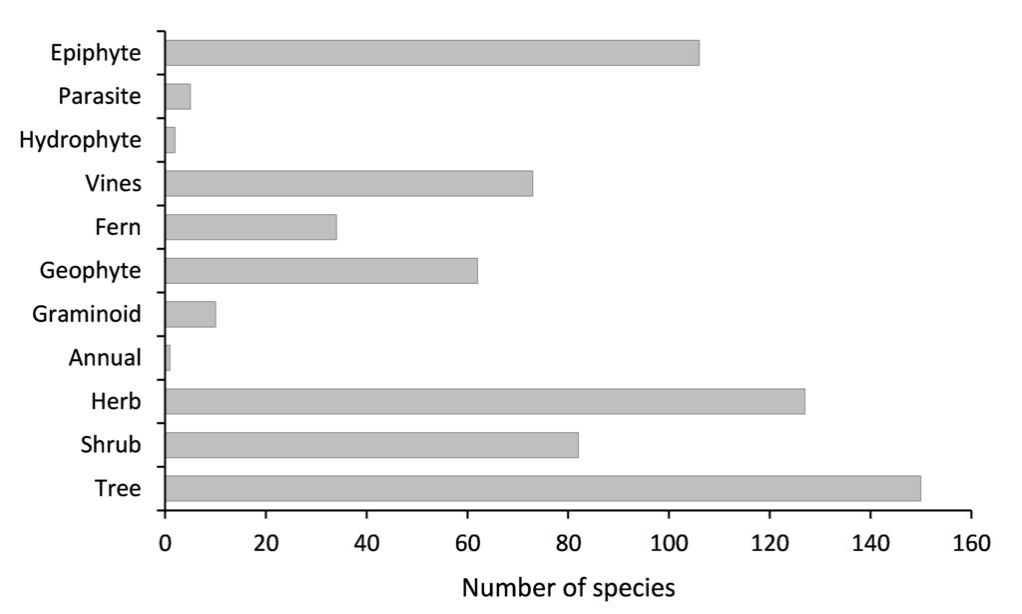
Species richness classified by number of habits.

**Figure 3.**
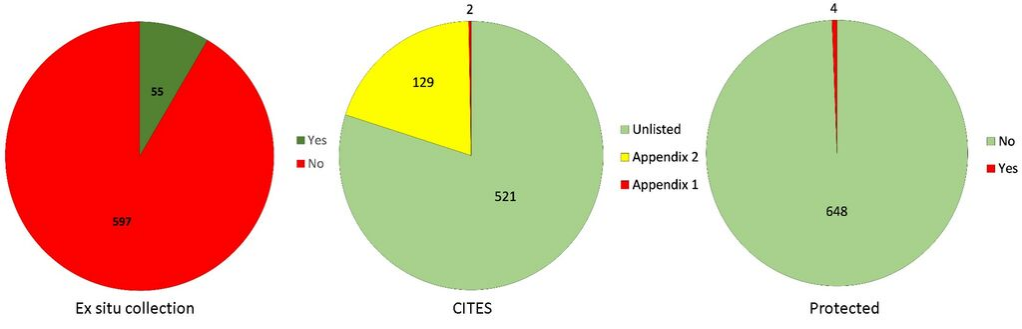
The number of Javan endemic plants that have been conserved (Yes) at *ex -situ* collections (left), listed on the CITES appendices (middle) and protected by the national law of Indonesia (right).

**Figure 4.**
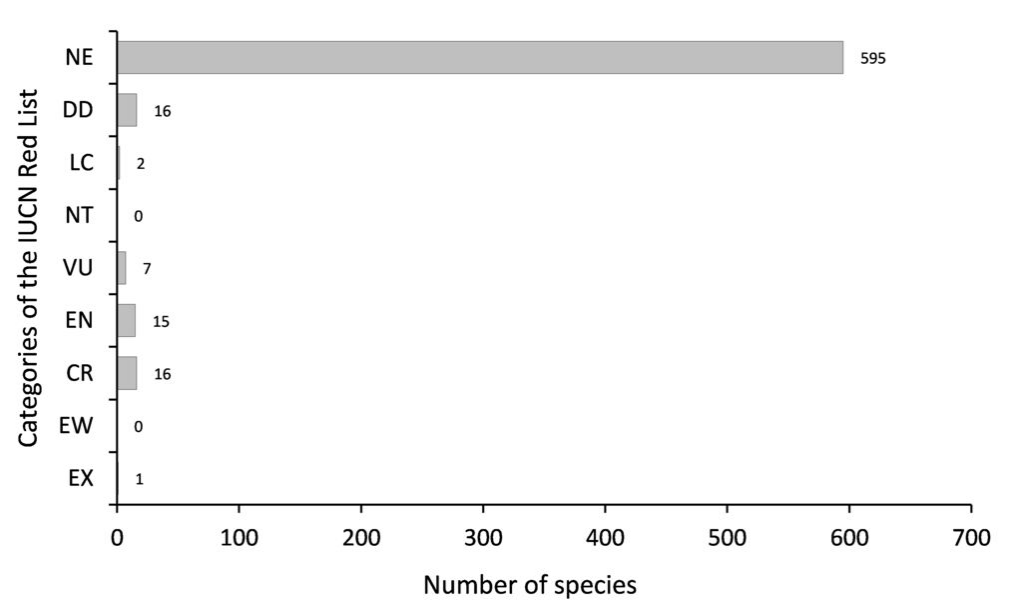
Conservation status of Javan endemic plants according to IUCN Red List Categories (EX = Extinct, EW = Extinct in the wild, CR = Critically endangered, EN = Endangered, VU = Vulnerable, NT = Near threatened, LC = Least concern, DD = Data deficient, NE = Not evaluated).

A list of species is essential for knowledge of species diversity in areas with strong anthropogenic pressure (Funk 2006, Hortal et al. 2007, Hermoso et al. 2013, Cornwell et al. 2018). It can be used to provide information for potential in situ and ex situ conservation planning efforts and as a baseline reference for future monitoring programmes. Therefore, in this contribution, we present a database of endemic plants of Java Island, Indonesia (Suppl. material 1). The data describes:

1. species composition (Fig. 1),
2. habit (tree, shrub, herb, annual, graminoid, geophyte, fern, vines, hydrophyte, parasite, epiphyte, Fig. 2),
3. ex situ collection status (Fig. 3), the protection status of the species according to
4. CITES (Fig. 3) and
5. the national law of Indonesia (Fig. 3) and
6. conservation status of the endemic species according to the IUCN Red List (Fig. 4)

## General description

### Purpose

This contribution provides a digital, comprehensive, publicly available and updatable database on the endemic plants of Java Island, Indonesia. It is intended as a working database for compiling current knowledge of the Javan endemic plants in a systematic way. The list will be amended over time as taxonomies are revised and new discoveries are made. The database serves as a baseline overview of current knowledge of the Island's endemic plants, which can be developed and refined over time.

## Project description

### Study area description

The study site is the mainland of Java and its surrounding small islands, including Panaitan Island in the westernmost, Kepulauan Seribu, Karimun Jawa, Bawean, Madura and Kangean Islands in the easternmost areas. We exclude Bali Island in the present study. Java Island has two contrasting climatic patterns: a wet tropical climate in the western part and a drier climate in the eastern part, where the dry season can last for four to six consecutive months (Fontanel and Chantefort 1978). According to Backer and Bakhuizen van den Brink (1965), the forests in Java show strong altitudinal gradients, including lowland forest (< 1000 m a.s.l.), lower montane forest (1000-1600 m a.s.l.) and upper montane forest (1500-2400 m a.s.l.) which is followed by the sub-alpine forest.

The plant diversity of Java has been well studied and described (e.g. Backer and Bakhuizen van den Brink 1968, van Steenis 1972) compared to that of the other islands in Indonesia. It has the highest collection density with 199 collections/100 km^2^, followed by Sulawesi (24/100 km^2^) and Sumatra (22/100 km^2^) (Middleton et al. 2019). The Flora of Java, published by Backer and Bakhuizen van den Brink in 1965-1968, is the first published vascular plant checklist of a large Malesian island.

## Sampling methods

### Sampling description

An initial dataset was searched and manually extracted from the Plants of the World Online (POWO 2021), an open and downloadable plant database with many interesting features, including: i) the backbone taxonomy of POWO is based on the World Checklist of Vascular Plants (WCVP 2022), which lists the known synonyms for each accepted species, ii) the database is updated weekly and reviewed by experts and iii) accepted species are associated with distribution data following the international standard of the World Geographical Scheme for Recording Plant Distributions (WGSRPD). We used a geographic search using the term “location: Jawa” and restricted the search to the species level with accepted names. Jawa is the Level-3 name for Java Island in the WGSRPD (Brummitt et al. 2001), the international standard used by POWO for recording plant distributions. A total of 6180 species resulted from this step. The distribution of each species in the resultant list was checked. We retained species with distribution only in Java and removed all the species that are present outside of the study area. The resulting list from this step contained 839 species names. We verified the endemicity of each species by checking the collection localities of its herbarium images stored in the Global Biodiversity Information Facility (GBIF 2021) and checking its records in the Flora of Java (Backer and Bakhuizen van den Brink 1968), the online database of the Catalogue of Life (COL 2021), taxonomic revisions, published checklists and peer-reviewed scientific papers. This final step produced a total of 652 endemic species of Java Island presented in this contribution. Family names and synonyms of the species were fully adopted from POWO (2021). We classified the conservation status and growth forms of each species according to the IUCN Red List (IUCN 2021) and the IUCN Plant Growth Forms Classification Scheme (IUCN 2020), respectively. Information on the possible ex situ conservation status of each species was obtained from PlantSearch! (BGCI 2021). We also verified the protection status of the species according to CITES (Appendices | CITES) and the national law of Indonesia Permen LHK P.106 2018 (Jaringan Dokumentasi Dan Informasi Hukum Kementerian Lingkungan Hidup Dan Kehutanan (menlhk.go.id)

## Geographic coverage

### Description

The study area is Java and its surrounding small islands (104°41′-116°01′E and 5°58′-8°50′S). It covers an area of 129,438.28 km^2^, bounded by the Indian Ocean in the south and the Java Sea in the north and lying between the Bali Strait to the east and the Sunda Strait to the west.

### Coordinates

Indian Ocean and Java Sea Latitude; Sunda Strait and Bali Strait Longitude.

## Traits coverage

Tree, Shrub, Herb, Annual, Graminoid, Geophyte, Fern, Vines, Hydrophyte, Parasite, Epiphyte.

## Temporal coverage

### Notes

2020-2022

## Usage licence

### Usage licence

Creative Commons Public Domain Waiver (CC-Zero)

## Data resources

### Data package title

Endemic plants of Java Island, Indonesia: a dataset.

### Number of data sets

1

### Data set 1.

#### Data set name

Endemic plants of Java Island, Indonesia: a dataset.

#### Data format

csv

#### Data format version

csv

#### Description

The dataset provides a list of 652 endemic species of Java Island, Indonesia. It describes: (i) family, (ii) synonyms, (iii) local name of the species (if available), (iv) habit (tree, shrub, herb, annual, graminoid, geophyte, fern, vines, hydrophyte, parasite, epiphyte), (v) ex situ collection status, (vi) conservation status of the species according to the IUCN Red List and the protection status of the species according to (vii) CITES and (viii) the national law of Indonesia.

**Data set 1.** 

Column label	Column description
dwc:scientificName	Scientific name of species and authority.
dwc:family	Family of the species.
synonym	Synonym of the species.
dwc:vernacularName	Local name of the species (if available).
habit	Growth habit according to literature. Tree, Shrub, Herb, Annual, Graminoid, Geophyte, Fern, Vines, Hydrophyte, Parasite, Epiphyte.
exSitu	Information on the possible ex situ collection status of species. Yes/No
dwc:threatStatus	Assignation the extinction risk of species according to the IUCN Red List Categories and Criteria. EX (Extinct), CR (Critically endangered), EN (Endangered), VU (Vulnerable), LC (Least concern), DD (Data deficient), NE (Not evaluated).
dwc:appendixCITES	Status of species according to CITES appedices: No (Unlisted), 1 (Appedix 1), 2 (Appendix 2).
protected	The protection status of species according to the national law of Indonesia: Yes (protected), No (unprotected).

## Supplementary Material

67D2A4A6-BB13-57E0-8554-8551677F6C16 10.3897/BDJ.10.e84303.suppl1 Supplementary material 1 Endemic plants of Java Island, Indonesia: a dataset Data type Species list Brief description The dataset provides a list of 652 endemic species of Java Island, Indonesia. It describes: (i) family, (ii) synonyms, (iii) local name of the species (if available), (iv) habit (tree, shrub, herb, annual, graminoid, geophyte, fern, vines, hydrophyte, parasite, epiphyte), (v) ex situ collection status, (vi) conservation status of the species according to the IUCN Red List and the protection status of the species according to (vii) CITES and (viii) the national law of Indonesia. File: oo_703551.tsv https://binary.pensoft.net/file/703551 Dipta Sumeru Rinandio, Hendra Helmanto, Rizmoon Nurul Zulkarnaen, Enggal Primananda, Arief Hamidi, Iyan Robiansyah
